# Flow-FISH as a Tool for Studying Bacteria, Fungi and Viruses

**DOI:** 10.3390/biotech10040021

**Published:** 2021-10-11

**Authors:** Julian J. Freen-van Heeren

**Affiliations:** Independent Researcher, Amsterdam, The Netherlands; j.freenvanheeren@gmail.com

**Keywords:** bacteria, DNA, Flow-FISH, fungi, protein, RNA, viruses, flow cytometry

## Abstract

Many techniques are currently in use to study microbes. These can be aimed at detecting, identifying, and characterizing bacterial, fungal, and viral species. One technique that is suitable for high-throughput analysis is flow cytometry-based fluorescence in situ hybridization, or Flow-FISH. This technique employs (fluorescently labeled) probes directed against DNA or (m)RNA, for instance targeting a gene or microorganism of interest and provides information on a single-cell level. Furthermore, by combining Flow-FISH with antibody-based protein detection, proteins of interest can be measured simultaneously with genetic material. Additionally, depending on the type of Flow-FISH assay, Flow-FISH can also be multiplexed, allowing for the simultaneous measurement of multiple gene targets and/or microorganisms. Together, this allows for, e.g., single-cell gene expression analysis or identification of (sub)strains in mixed cultures. Flow-FISH has been used in mammalian cells but has also been extensively employed to study diverse microbial species. Here, the use of Flow-FISH for studying microorganisms is reviewed. Specifically, the detection of (intracellular) pathogens, studying microorganism biology and disease pathogenesis, and identification of bacterial, fungal, and viral strains in mixed cultures is discussed, with a particular focus on the viruses EBV, HIV-1, and SARS-CoV-2.

## 1. Introduction

A myriad of techniques is available for the detection, identification, and characterization of bacterial, fungal, and viral species. Most commonly, these techniques probe the genome of microorganisms, i.e., sequencing of 16S ribosomal RNA, to identify bacterial species [1]. However, these techniques are often unable to provide information regarding the relative abundance, or, in the case of intracellular microorganisms, the percentage of infected cells.

Ideally, a single-cell approach is used for the detection and characterization of (intracellular) microorganisms. One technique that is suitable for this purpose in a high-throughput fashion is flow cytometry-based fluorescence in situ hybridization (Flow-FISH) [2,3,4]. This technique employs highly specific probes directed against DNA or (m)RNA specific to the transcript or (microorganism) species of interest. These probes can be directly labeled with a fluorophore but are sometimes also visualized through sequential binding steps with, e.g., biotin and streptavidin. Flow-FISH can also be multiplexed, allowing for measuring several RNA species [5]. Furthermore, Flow-FISH assays can also be combined with fluorescently labeled antibodies [6]. When targeting mRNA, this allows for the concomitant measurement of mRNA and protein of the same gene [3,5,6]. Of note, Flow-FISH has recently even been employed for the cell sorting of live bacteria [7,8].

By making use of (online) tools (e.g., the Stellaris Probe Designer by Biosearch Technologies), probe set design is straightforward. Due to this relatively easy design process, Flow-FISH can be a valuable tool in research settings where no good (fluorescently labeled) antibodies are available for the target of interest (i.e., difficult to stain cytokines such as IL-21 [2]), when no protein product is formed (i.e., noncoding RNAs such as microRNAs [9,10]), or when studying (model) organisms for which the antibody toolbox has not yet been perfected or developed (i.e., fruit-eating bats [11]). Furthermore, Flow-FISH assays are more readily adaptable than antibody-based detection, making it extremely suitable to study rapidly mutating organisms such as viruses.

Flow-FISH has mainly been employed in three fields of study: (1) studying T cell biology and effector function [2,12,13,14], (2) assessing telomere length [15,16], and (3) detecting and studying (intracellular) bacteria, fungi, and viruses [17,18,19,20]. Here, the use of Flow-FISH for the detection of (intracellular) pathogens, studying microorganism biology and disease pathogenesis, and the identification of bacterial, fungal, and viral strains in mixed cultures is discussed, with a particular focus on studies investigating Epstein–Barr virus (EBV), human immunodeficiency virus 1 (HIV-1), and severe acute respiratory syndrome coronavirus 2 (SARS-CoV-2), the causative agent responsible for the coronavirus disease 2019 (COVID-19) pandemic.

## 2. General Principles and Brief Overview of Different Types of Flow-FISH Assays

In this review, the term Flow-FISH is used interchangeably for the measurement of genetic material (either DNA or RNA), either standalone, or in combination with (microbial) proteins. Furthermore, while Flow-FISH is used here as a broad term for this technique, several different types of Flow-FISH assays have been developed for flow cytometric detection of genetic information. The different techniques employed in microorganisms can be categorized into three main categories: (1) single-molecule Flow-FISH utilizing single probes, (2) single-molecule Flow-FISH utilizing multiple probes, and (3) Flow-FISH utilizing branched signal amplification. These different techniques have been elaborately discussed elsewhere [21], but their basic principles and applications will be discussed here briefly.

### 2.1. Single-Molecule Flow-FISH Utilizing Single Probes

One of the most employed types of Flow-FISH assay for the detection of microbial genetic information is single-probe Flow-FISH. This approach is suitable for the detection of DNA, microRNA, and (m)RNA [9,15,16,22,23,24]. As only one probe is used, single-probe Flow-FISH requires high sequence specificity and probe affinity to the target gene. Typically, locked nuclear acids (LNAs) and peptide nuclear acids (PNAs) are used in this type of Flow-FISH assay. LNAs and PNAs are more stable than the typical DNA-based probes used in, e.g., single-molecule Flow-FISH utilizing multiple probes. Therefore, LNA and PNA probes allow for higher hybridization temperatures, enhancing probe specificity. LNA and PNA probes can be directly fluorescently labeled, but biotin-labeled variants have also been employed. Subsequent fluorescently labeled streptavidin can then be used to visualize the probe, further amplifying the signal. However, at the same time, this also increases the background noise. Of note, when studying telomeres, this type of Flow-FISH is most often used [16].

### 2.2. Single-Molecule Flow-FISH Utilizing Multiple Probes

Another Flow-FISH assay that is often employed is single-molecule Flow-FISH with multiple probes. Typically, these probes are 20-nucleotide-long single-strand oligo (DNA) nucleotides, suitable for the detection of microRNA and (m)RNA [5,13,25]. These probe sets, which are custom-designed for the target or microorganism of interest, can be purchased directly labeled on either the 5′ or 3′ end, or can be custom-labeled in-house [26] for inclusion in established flow cytometry panels based on investigator needs. Single-molecule Flow-FISH utilizing multiple probes can be combined with protein measurements [5,12,14], and can be multiplexed [5].

### 2.3. Flow-FISH Utilizing Branched Signal Amplification

Several commercial suppliers also offer Flow-FISH kits suitable for the detection of DNA, mRNA, and microRNA [2,27]. Generally, these kits amplify the signal through consecutive incubations. This type of Flow-FISH assay makes use of DNA-based probes that contain overhangs. In subsequent hybridization steps, these overhangs are bound by secondary probes. A tertiary labeled probe is then used for fluorescent detection, ultimately multiplying the original probe signal >100 times. This renders this technique highly suitable to detect genomic information at low copy numbers, but also for the measurement of small RNAs, such as microRNAs [2]. While commercial kits can be used off-the-shelf, relatively, they are more expensive. Furthermore, the serial amplification steps are time consuming. Of note, similar to single-molecule Flow-FISH utilizing multiple probes, branched signal amplification can also be combined with protein staining and can be multiplexed [2].

## 3. Flow-FISH Applications in Microorganisms

Flow-FISH has been employed to study bacteria, fungi, and viruses. Especially for research in viral pathogens such as EBV and HIV-1, Flow-FISH has been a valuable tool to investigate, e.g., viral latency and disease pathology. Here, the use of Flow-FISH in each clade is briefly discussed, with a specific focus on several intensively studied viruses, or viruses of particular interest.

### 3.1. Bacteria

Flow-FISH has been employed for diverse applications in several different bacterial species, such as *Escherichia coli* and *Clostridium* species (Table 1). If available, probe sequences and limits of detection can be found in Supplementary Table S1. By using probes directed at specific bacterial sequences, individual bacterial species can be easily identified [17,28,29,30,31,32,33], preventing the need for, e.g., extensive culturing. Similarly, causative agents in bacterial infections [34,35] or food contamination [34,35,36,37,38] can also be more easily identified by using Flow-FISH. Especially in diagnostic settings, the speed advantage of Flow-FISH, which typically takes 1–2 days, compared to, e.g., culturing could be very beneficial. Furthermore, in contrast to other techniques, such as culturing or 16S ribosomal RNA sequencing, Flow-FISH provides information on the relative abundance. On the other hand, only species that have been specifically targeted with probes can be detected, while, e.g., 16S sequencing provides information on most, if not all, present microorganisms.

Flow-FISH has also been used to study bacterial noncoding RNA expression [9,10], marine bacteria [33], and for the cell sorting of live bacteria [7,8]. In particular, the recent development of a live sorting technique for bacteria via Flow-FISH could prove very useful in the future, where pure bacterial isolates and/or single-cell-derived cultures could be achieved more easily. Other potential applications include sorting of live bacteria based on gene expression to investigate the role of gene(s) of interest on, e.g., growth patterns, metabolite usage, bacterial virulence, and/or antibiotic resistance.

### 3.2. Fungi

Flow-FISH assays have been employed to study the fungal strains *Candida albicans*, *Saccharomyces carlsbergensis*, *Staphylococcus aureus,* and *Staphylococcus epidermidis* (Table 2). If available, probe sequences and limits of detection can be found in Supplementary Table S2. These Flow-FISH assays were set up to detect fungal strains [17,18,39], assess food contamination [35], investigate ex vivo infection models [39,40,41], and to study antimycotic resistance [42]. Similar to bacterial Flow-FISH assays, also in fungal microorganisms, Flow-FISH offers the advantage of providing information on relative abundance.

**Table 1:** Studying bacteria with Flow-FISH.

Species	Sample Type(s)	Application	Reference
*Bacillus cereus*	Collection strain	Strain identification	[28]
*Bacteroides vulgatus*	Fecal sample	Strain identification	[43]
*Bifidobacterium longum*	Fecal sample	Strain identification	[43]
*Carnobacterium* spp.	Collection strain	Strain identification	[29]
*Clostridium* spp.	Fermentative culture	Strain identification	[30]
*Collinsella aerofaciens*	Fecal sample	Strain identification	[43]
*Desulfovibrio gigas*	Collection strain	Strain identification	[31]
*Desulfobacter hydrogenophilus*	Collection strain	Strain identification	[31]
*Escherichia coli*	Collection strain	Growth pattern analysis Strain identification	[17]
	Collection strain	Strain identification	[31]
	Collection strain	Strain identification	[33]
	Collection strain	Strain identification	[44]
	Ex vivo infected blood	Detection of infection	[34]
	Fecal sample	Strain identification	[43]
	Lab-infected food (milk)	Food contamination	[35]
*Faecalibacterium prausnitzii*	Fecal sample	Strain identification	[43]
*Klebsiella pneumoniae*	Ex vivo infected blood	Detection of infection	[34]
*Lactobacillus brevis*	Collection strain	Strain identification	[29]
*Pseudomonas* spp.	Collection strain	Growth pattern analysis Strain identification	[17]
	Collection strain	Strain identification	[44]
	Ex vivo infected blood	Detection of infection	[34]
	Food (milk)	Food contamination	[32]
	Lab-infected food (milk)	Food contamination	[35]
*Ruminococcus productus*	Fecal sample	Strain identification	[43]
*Salmonella* spp.	Food (tomato)	Food contamination	[36]
	Food (tomato)	Food contamination	[37]
	Food (alfalfa)	Food contamination	[38]

While not yet employed on patient samples, Flow-FISH could be valuable as a tool to detect causative pathogens in sepsis patients, potentially leading to more targeted therapeutic strategies. However, as time is essential in the treatment of (fungal) sepsis patients, more protocol optimization is required if Flow-FISH is to be used as a diagnostic tool, as most Flow-FISH assays incorporate (at least) an overnight incubation step. Other future applications where Flow-FISH could be used to study fungal species include studying gene transcription/single-cell gene expression based on stimuli or growth conditions under diverse conditions, or in antimycotic research. 

### 3.3. Viruses

While Flow-FISH has been employed to study both bacteria and fungi, it has mostly been used as a tool to study a variety of viruses. These include both human pathogens, such as dengue virus or Zika virus [45], and pathogens that also propagate in mice (yellow fever virus [46,47]) or specifically propagate in nonhumans, such as cattle (bovine viral diarrhea virus [48,49]) or simians (simian varicella virus [50]). These applications include the detection of infected cells [45,51], viral strain identification [49], and studying viral biology [45,46,47]. An overview of studies employing Flow-FISH assays to study viruses can be found in Table 3. If available, probe sequences and limits of detection can be found in Supplementary Table S3. The viruses that have been most studied with Flow-FISH (EBV and HIV-1) or viruses of specific interest (SARS-CoV-2) are next discussed in more detail.

#### 3.3.1. EBV

EBV is an oncogenic gamma herpesvirus that selectively infects humans, most commonly targeting B lymphocytes and epithelial cells. EBV is best known as the causative agent of infectious mononucleosis. After infection, EBV persists as an asymptomatic latent infection in immunocompetent individuals, while in immunocompromised subjects, EBV infection is associated with life-threatening pathologies [52]. Furthermore, it is suspected that EBV plays a role in a range of cancers, including B cell neoplasms and nasopharyngeal carcinomas, contributing to approximately 1.5% of cancers in humans worldwide [53].

**Table 3:** Studying viruses with Flow-FISH. BVDV, bovine viral diarrhea virus; γHV, gamma herpesviruses; KSHV, Kaposi’s sarcoma-associated herpesvirus; SV, Sindbis virus; SVV, simian varicella virus; YFV, yellow fever virus.

Species	Cell Type(s)	Application	Reference
BVDV	Bovine lymphoid cells	Detection of infected cells	[48]
	Cell lines	Viral strain identification	[49]
Dengue virus	Cell lines	Detection of infected cells	[45]
HCV	Cell lines	Detection of infected cells	[54]
γHV	Cell lines	Detection of infected cells	[55]
KSHV	Cell lines	Detection of infected cells	[56]
	Cell lines	Detection of infected cells	[55]
Parvovirus B19	Cell lines	Detection of infected cells	[57]
	Erythroid progenitor cells	Detection of infected cells Parvovirus B19 biology	[51]
Poliovirus	Cell lines	Detection of infected cells Poliovirus biology	[45]
SV	In vitro model	Detection of infected cells	[22]
SVV	Cell lines	Detection of infected cells	[50]
YFV	Cell lines Murine PBMC	Detection of infected cells YFV biology	[46]
	Cell lines	YFV biology	[47]
Zika virus	Murine leukocytes	Detection of infected cells	[58]
	Cell lines	Zika biology	[45]

Therefore, understanding EBV biology is of paramount importance. Flow-FISH has been a valuable tool in EBV research (Table 4). If available, probe sequences and limits of detection can be found in Supplementary Table S4. For instance, Flow-FISH was used to study EBV gene expression in EBV-infected cell lines [55,59,60,61,62]. By spiking an EBV^+^ cell line into an EBV^-^ cell line, the EBV Flow-FISH assay was determined to be sensitive enough to detect up to ~0.01% EBV^+^ cells (approximately 1 in 10,000) [61]. Furthermore, Flow-FISH has also been used to show that, even in cell lines, which are most often considered semi-synchronous cultures, viral RNA expression showed cell-to-cell variation [55]. This highlights the single-cell advantage of Flow-FISH over more commonly used (diagnostic) methods such as (q)PCR.

EBV Flow-FISH has also been employed in patients with lymphoproliferative diseases [61,63]. While the EBV Flow-FISH assay did not identify any cells positive for EBV DNA in peripheral blood cells obtained from healthy individuals, four out of four patients with lymphoproliferative disease showed varying degrees of EBV^+^ cells, with one hydroa vacciniforme-like patient exhibiting 25.9% EBV^+^ lymphocytes in circulation [61]. As Flow-FISH is a flow cytometric assay, the types of infected cells could also easily be determined. By making use of fluorescent antibodies against surface markers, authors were able to identify that the majority of EBV-infected cells in the aforementioned patient were CD3^+^TCRγδ T cells [61]. This was confirmed through conventional qPCR after cell sorting, showing a correlation between the EBV Flow-FISH assay and conventional diagnostic methods [61]. Similarly, a recent study by Fournier et al. also utilized EBV Flow-FISH for the characterization of infected cell types [62]. In individuals with severe infectious mononucleosis, B cell lymphoproliferative disease and several patients with primary immunodeficiencies, CD19^+^ B cells were shown to be the main EBV DNA-expressing population [62]. In contrast, in patients with T cell or NK cell lymphoproliferative diseases, the major EBV DNA-expressing population were CD3^+^ T cells [62]. The EBV Flow-FISH assay was also successfully employed to assess EBV DNA in T cells of patients with suspected EBV-mediated T/NK cell lymphoproliferative disease [62], showing promise as a diagnostic tool. Lastly, in hydroa vacciniforme-like patients, the majority of EBV-infected cells were identified as CD3^+^TCRγδ T cells by Fournier et al. [62], confirming previously reported data [61,63].

The cellular and molecular characteristics of EBV-infected cells are also still largely unknown. By making use of the EBV Flow-FISH assay, Fournier et al. were able to show that EBV-infected B cells are largely IgD^−^, indicative of antigen-experienced B cells, and could generally be identified as germinal center B cells and plasma cells based on CD19, CD21, CD27, CD38, and IRF4 expression [62]. Additionally, Flow-FISH analysis showed that the majority of EBV-infected T cells were effector memory T cells based on CD27 and CD45RA expression, and expressed HLA-DR. In contrast, noninfected cells did not show an enrichment for any specific T cell population, and the amount of HLA-DR-expressing noninfected cells was also lower [62]. This extensive phenotyping of EBV-infected primary cells from patients could, in future, potentially lead to new insights or novel therapeutic strategies.

Together, these reports show the advantage of a flow cytometric read-out in EBV-infected individuals, and also show that Flow-FISH can aid in diagnosis of EBV-related malignancies. Through the combination of EBV DNA detection with Flow-FISH and fluorescent antibodies targeting surface antigens, the main types of infected cells can be identified and quantified. This eliminates the need for cell sorting. Furthermore, cell sorting is never 100% accurate. Therefore, it could potentially result in false positives [62], and ultimately lead to misdiagnosis of the type of malignancy. Therefore, Flow-FISH could be a valuable (additional) tool in diagnosis of (suspected) EBV-mediated lymphoproliferative disorders. Of note, as shown by Fournier et al. [62], Flow-FISH can also be used to study EBV biology, e.g., by extensive phenotyping of preferentially infected cells. Other applications include, e.g., studying latent and lytic infection cycles, or the influence of EBV gene expression on, e.g., host cell growth or apoptosis.

#### 3.3.2. HIV-1

HIV-1, a retrovirus, was described in 1983 as the causative agent of acquired immunodeficiency syndrome, or AIDS [65]. Since then, the diagnosis, treatment, and monitoring of AIDS patients has progressed massively [4,20,66,67]. With the advent of antiretroviral therapy, HIV-1 propagation can be kept in check, preventing the development of AIDS. Instead, HIV-1-infected individuals experience a latent infection, in which HIV-1 rarely propagates. However, antiretroviral therapy has toxic side events and can impact life expectancy. Currently, a definitive cure for HIV-1 infection is not available. However, over the years, more information critical for designing therapeutic approaches has been garnered. For instance, recent years have seen an increase in knowledge regarding the type of cells harboring latent HIV-1 infection [6] due to the rise of novel single-cell technologies, including Flow-FISH. The use of single-cell technologies in HIV-1 research has recently been reviewed elsewhere [68]. An overview of studies investigating HIV-1 in which Flow-FISH assays were employed can be found in Table 5. If available, probe sequences and limits of detection can be found in Supplementary Table S5.

In general, the studies employing HIV-1 Flow-FISH can be divided in four categories: (1) using Flow-FISH to detect infected cells, (2) studying HIV-1 biology, (3) investigating the latent viral reservoir, divided into the translation-competent and replication-competent viral reservoir, and (4) latency reversal research.

Firstly, Flow-FISH can simply be used to detect HIV-1-infected cells. Indeed, HIV-1 Flow-FISH assays have been employed to study infected cell lines [69], and ex vivo infected cells, such as total PBMCs [70], epidermal DCs [71], and T cells [72,73], but also patient-derived material, such as alveolar macrophages [74], T cells [3,19,74,75], and even platelets [75]. In the future, Flow-FISH might even be useful as a tool to monitor therapy effectiveness and assessment of total eradication of (latently) infected cells [20].

HIV-1 Flow-FISH can also be of interest when studying HIV-1 biology. For instance, Flow-FISH was used to investigate cellular characteristics of HIV-1-infected T cells. In ex vivo infected PBMCs and cells from HIV-1-infected patients, cells that are actively transcribing HIV-1 RNA are enriched for memory T cells compared to nontranscribing T cells [76]. Similarly, in untreated HIV-1-infected individuals, p24-producing cells (indicative of cells that are translationally competent) are enriched for memory T cells, peripheral T follicular helper cells, and regulatory T cells [6]. Furthermore, more p24-producing cells expressed the activation markers CD38, CD69, and HLA-DR, more p24-producing cells stained positive for Ki67, a marker for proliferating cells, and more p24-producing cells expressed the inhibitory receptors LAG-3, TIM-3, PD-1, and TIGIT, compared to noninfected CD4^+^ T cells [6]. In addition, in patients on antiretroviral therapy, p24-producing cells were enriched for memory T cells, and also here the frequency of cells that expressed PD-1 and TIGIT was significantly higher in p24-producing cells compared to nonproducing cells [6].

**Table 5:** Studying HIV-1 with Flow-FISH.

Cell Type(s)	Application	Reference
Cell lines	Detection of infected cells	[69]
Ex vivo infected PBMCs	Detection of infected cells	[70]
Ex vivo infected epidermal DCs	Detection of infected cells	[71]
Ex vivo infected T cells	Detection of infected cells Anti-HIV antibody biology	[72]
Patient T cells	Detection of infected cells	[3]
Patient T cells Patient alveolar macrophages	Detection of infected cells	[74]
Patient T cells	Detection of infected cells Studying HIV biology	[77]
Patient T cells	Detection of infected cells Studying HIV biology	[19]
Cell lines Ex vivo infected T cells	Latency reversal Host antiviral factors	[73]
Patient T cells	Studying HIV biology Translation-competent viral reservoir	[78]
Ex vivo infected T cells Patient T cells	Studying HIV biology Translation-competent viral reservoir	[76]
Patient T cells	Studying HIV biology Translation-competent viral reservoir	[6]
Patient T cells	Translation-competent viral reservoir	[79]
Patient platelets	Replication-competent viral reservoir	[75]
Cell lines Patient T cells	Latency reversal	[80]
Cell lines Patient T cells	Latency reversal	[81]
Cell lines Ex vivo infected T cells	Latency reversal	[82]

Flow-FISH analysis can also potentially lead to new therapeutic insights [20]. For instance, Flow-FISH was used to show that patient-derived HIV-1-infected T cells expressed CD20 upon viral reactivation [77]. CD20, typically a B cell marker, can be targeted with the monoclonal therapeutic antibody rituximab [83]. The low levels of CD20 expressed by HIV-1-infected T cells can render them sensitive to rituximab-mediated killing [77]. Similarly, Flow-FISH revealed that HIV-1-infected T cells that actively transcribe HIV-1 RNA, but not nontranscriptionally active HIV-1-infected T cells, express CD32 [19]. Both CD20 and CD32 have been explored as targets for CAR-T cellular therapy for other indications [84,85]. However, due to the limited projected therapeutic benefit and major (immunopathological) side effects that are to be expected, CAR-T therapy targeting CD20 or CD32 as a therapeutic for HIV-1 might not be optimal. Similarly, rituximab treatment for the treatment of HIV-1 would also result in significant off-target effects (i.e., removal of a patient’s B cell compartment).

HIV-1 Flow-FISH has also been employed to study antiviral host factors. For instance, Flow-FISH was used to detect viral RNA in cell lines with deletions in the viral surveillance proteins UPF1, UPF2, and SMG6 [73]. Authors showed that UPF1, normally considered an antiviral host factor, is a positive regulator of HIV-1 reactivation. Indeed, UPF1 deletion resulted in impaired viral RNA expression, while UPF1 overexpression enhanced viral RNA expression [73]. In contrast, UPF2 and SMG6 were identified as host factors negatively regulating HIV-1 RNA expression. Specifically, UPF2 and SMG6 were shown to interact with UPF1 and inhibit UPF1 function [73]. These results were subsequently also validated in HIV-1-infected primary CD4^+^ T cells [73].

Other researchers have employed HIV-1 Flow-FISH assays to investigate anti-HIV-1 antibodies. The HIV-1 protein gp120 is shed from infected cells and can bind to CD4 expressed on the cell surface. It has been hypothesized that antibodies directed against gp120 could result in the unwanted killing of noninfected healthy CD4^+^ T cells [86]. To investigate this, authors performed binding assays of anti-HIV-1 antibodies in mixed cultures of noninfected CD4^+^ T cells and ex vivo HIV-1-infected CD4^+^ T cells [72]. They employed an antibody clone, A32, that can only interact with gp120 when it is bound to CD4, as the A32 binding site is occluded in non-CD4-bound gp120. By employing Flow-FISH, authors could differentiate cells expressing HIV-1 *GagPol* mRNA and staining positive for HIV-1 p24 protein (i.e., HIV-1-infected cells) from noninfected cells and showed an enrichment for noninfected CD4^+^ T cells in the A32-bound fraction. Together, these results confirm that the A32 antibody clone does not recognize infected cells, but indeed rather targets noninfected bystander cells.

Another main application of HIV-1 Flow-FISH assays is to study latent HIV-1 infection. As discussed, if properly treated with antiretroviral therapy, HIV-1 forms a latent infection cycle. Identifying and characterizing the latently infected cells and, perhaps even more importantly, the cells forming the translationally-competent and replication-competent reservoir could lead to new therapeutic strategies [20]. Firstly, identifying cells that form the latent reservoir could be instrumental for the eradication of these specific viral reservoirs. For instance, as discussed, HIV-1 T cells that are actively transcribing HIV-1 RNA were shown to express CD32 [19,76]. Interestingly, in patients treated with antiretroviral therapy, transcriptionally capable cells, as measured by cells producing HIV-1 p24 protein, do not express CD32 [6]. Of note, it was recently shown through Flow-FISH analysis that even platelets can harbor latent replication-competent HIV-1 virus [75].

The translation-competent reservoir also impacts HIV-1-specific T cell responses [79]. In a recent article, Niessl et al. investigated the correlation between HIV-1 specific T cells and the production of effector cytokines and expression of inhibitory molecules. Not surprisingly, the expression of inhibitory receptors such as PD-1 and TIGIT by HIV-1-specific T cells correlated with the size of the translation-competent reservoir. Interestingly, even though these cells are continuously exposed to antigen, the size of the translation-competent reservoir also positively correlated with the production of effector molecules such as interferon γ and tumor necrosis factor α by T cells.

Thus, Flow-FISH has allowed for characterizing latently infected cells and investigating how latent HIV-1 infection impacts immune responses in general. This new information contributes to the development of new treatment strategies.

HIV-1 Flow-FISH assays have also been employed as a tool to study HIV-1 (pro)virus production upon cellular activation, or latency reversal [76,78,79,82]. This can be achieved by using cellular activators, including chemicals such as PMA and ionomycin [78], but also a DDX3 inhibitor was shown to reverse HIV-1 latency [81]. Hypothetically, reversing HIV-1 latency results in antigen presentation by infected cells. By harnessing immune cells, infected cells could potentially be targeted through enhancement of naturally occurring anti-HIV-1 (cellular) immune responses, or by making use of adoptive cellular therapies [20].

Interestingly, inhibition of DDX3 did not only reverse HIV-1 latency. Indeed, DDX3 inhibitors were also shown to selectively result in cell death of HIV-1 RNA-expressing cells in vitro [81]. Furthermore, through consecutive rounds of in vitro culture of HIV-1-infected cells in the presence of DDX3 inhibitors, authors showed that DDX3 inhibition resulted in a reduction of the viral reservoir that could be induced to transcribe viral RNA [81], providing proof-of-concept for the pharmacological reversal and eradication of latently infected cells. However, as this is preclinical data, much work is still required before DDX3 inhibitors can be applied in therapeutic applications in patients.

Other potential areas of HIV-1 research could also benefit from Flow-FISH. For instance, Flow-FISH could be employed to investigate viral spreading through antigen-presenting cells, a subset of cells that has also been shown to harbor and spread HIV-1 viral particles [87]. Furthermore, Flow-FISH could be used to monitor the cells harboring viral reservoirs, for instance during T cell or CAR-T cell therapy directed against HIV-1 antigen-expressing cells.

Flow-FISH has contributed to diverse fields in HIV-1 research. By making use of single-cell technologies such as Flow-FISH, new insights into HIV-1-mediated pathology, disease biology, and potential therapeutic strategies can be gained.

#### 3.3.3. SARS-CoV-2

Flow-FISH is also suitable to investigate emerging and novel viruses such as dengue virus or Zika virus [45]. Recently, Flow-FISH was applied to study the novel SARS-CoV-2 pathogen [88]. SARS-CoV-2 is a single-strand RNA virus and is the causative agent of COVID-19. This easily transmittable respiratory virus mainly causes respiratory problems and has, at the time of writing, infected more than 230 million people worldwide (https://covid19.who.int/ accessed on 3 October 2021).

Studying SARS-CoV-2 with fluorescence microscopy has been recently discussed elsewhere [89]. However, FISH and Flow-FISH have also been employed to study SARS-CoV-2 [88,90] (see Table 6). If available, probe sequences and limits of detection can be found in Supplementary Table S6. In a recent unreviewed preprint, FISH was used to detect SARS-CoV-2 with a probe set that targeted the conserved regions of the SARS-CoV-2 genome [90]. Authors were able to detect SARS-CoV-2 mRNA in cell lines, postmortem patient tissue samples, and nasal swabs typically used for SARS-CoV-2 diagnostic purposes. By employing FISH in cell lines, authors set up a proof-of-principle drug-screening test in which they showed that, in contrast to nontreated cells, cells pretreated with remdesivir, an antiviral agent, were not susceptible to SARS-CoV-2 infection. This approach could potentially be used to study other (prospective) antiviral drugs. However, as only one postmortem sample and one nasal swab was tested, more research is required to determine whether this method is robust enough for the detection of SARS-CoV-2 in primary patient samples. Please note that, as this publication is currently in preprint, authors might still address these concerns in a peer-reviewed version. Furthermore, microscopic RNA assessment and analysis FISH-data is labor-intensive, where the labor burden and required expertise required for Flow-FISH is lower. Therefore, Flow-FISH might be more suitable for studying virus biology, antiviral drugs, and potential diagnostic purposes.

SARS-CoV-2 Flow-FISH has already been set up and employed to investigate the role of an SHMT1/2-specific dual inhibitor on susceptibility of A549 cells, a human alveolar basal epithelial cell line, in SARS-CoV-2 infection [88]. SHMT1 and SHMT2 are metabolic enzymes that play a role in one-carbon folate species generation. The employed SARS-CoV-2 Flow-FISH assay showed that the SHMT1/2-inhibitor treatment significantly diminished SARS-CoV-2 nucleocapsid RNA expression in infected cells [88], hinting that folate species could potentially be a therapeutic target in future SARS-CoV-2 outbreaks.

Telomere Flow-FISH, another often-applied Flow-FISH assay, has also been employed to understand SARS-CoV-2 pathology. Authors found that short leucocyte telomeres were correlated with increased risk of severe COVID-19 [91]. However, as telomeres shorten during aging [92], and higher age groups are at increased risk of COVID-19-related mortality [93], these results are not very surprising. Of note, a recent, unreviewed preprint also employed telomere Flow-FISH to study SARS-CoV-2, and showed that telomeres are of comparable length in both COVID-19 patients and age-matched controls, indicating that no increased cellular attrition occurs in COVID-19 patients [94]. Of note, this publication is currently a preprint, and thus conclusions might still change in the peer-reviewed version.

Especially in emerging diseases, where traditional fluorescent detection tools such as antibodies have not yet been produced and/or manufactured, Flow-FISH can be extremely suitable, as is indicated by the studies discussed here. While not broadly employed (yet) to study SARS-CoV-2, the ease of probe-set design and single-cell approach can be a major benefit in unraveling the biology and pathology of recently discovered pathogens.

## 4. Conclusions and Outlook

Here, the myriad applications of Flow-FISH to detect and study diverse microorganisms have been discussed. Due to its single-cell approach, Flow-FISH assays provide more information compared to conventional diagnostic tests. In fact, Flow-FISH has even been suggested as a tool for clinical and/or diagnostic applications in HIV-1 therapy [20]. However, as discussed, the execution of a Flow-FISH assay requires trained personnel and can be more time consuming than traditional diagnostic testing [20], which should be carefully considered before implementing Flow-FISH in routine diagnostics. In contrast, as a research tool, Flow-FISH has broad applications in diverse microbiology fields. It can be employed for food safety [35], studying basic microbial and pathogen biology [6], and has even been used as a tool to investigate antimicrobial agents [42]. Furthermore, due to the ease of probe-set design, Flow-FISH has also been a useful tool in studying pathogens responsible for emerging diseases, such as dengue virus, SARS-CoV-2, and Zika virus [45,88].

Flow-FISH also has potential for new applications. For instance, combined with genetic knockouts, Flow-FISH could be used to study the effect of knockouts on gene expression at a single-cell level, or the importance of the expression of relevant genes in antibiotic/mycotic resistance. The recent development of a Flow-FISH-based live cells sorting technique for bacteria [7,8] also allows for sorting cells based on gene expression to, e.g., identify genes important in cell growth, division of cellular metabolism, or microorganism adaptability based on new environments or response to stimuli.

Therefore, while being time consuming, the single-cell approach and independence from traditional reagents required for flow cytometry make Flow-FISH a valuable tool to study microorganisms.

## Figures and Tables

**Table 2:** Studying fungi with Flow-FISH.

Species	Sample Type(s)	Application	Reference
*Candida albicans*	Clinical isolates	Fungal strain identification	[18]
	Collection strain Ex vivo infected blood	Fungal strain identification Detection of infection	[39]
*Saccharomyces carlsbergensis*	Collection strain	Fungal strain identification	[17]
*Staphylococcus aureus*	Ex vivo infected blood	Detection of infection	[40]
	Clinical isolates	Antimycotic resistance	[42]
	Lab-infected food (milk)	Food contamination	[35]
*Staphylococcus epidermidis*	Collection strain Ex vivo infected blood	Fungal strain identification Detection of infection	[41]

**Table 4:** Studying EBV with Flow-FISH.

Cell Type(s)	Application	Reference
Cell lines	Detection of infected cells	[59]
Cell lines Patient primary cells	Detection of infected cells	[60]
Cell lines Patient primary cells	Detection of infected cells EBV-mediated pathologies	[61]
Cell lines	Detection of infected cells	[55]
Cell lines Patient primary cells	Detection of infected cells EBV-mediated pathologies	[62]
Patient primary cells	Detection of infected cells EBV-mediated pathologies	[63]
Primary B cells	Detection of in vitro infected cells	[64]

**Table 6:** Studying SARS-CoV-2 with Flow-FISH.

Cell Type(s)	Application	Reference
Cell lines	Detection of infected cells SARS-CoV-2 biology and treatment Host factor–virus interaction	[88]

## Data Availability

Not applicable.

## Supplementary Materials

The following are available online at https://www.mdpi.com/article/10.3390/biotech10040021/s1, Supplementary Table S1. Sequences and limit of detection for bacterial Flow-FISH assays, Supplementary Table S2. Sequences and limit of detection for fungal Flow-FISH assays, Supplementary Table S3. Sequences and limit of detection for viral Flow-FISH assays, Supplementary Table S4. Sequences and limit of detection for EBV Flow-FISH assays, Supplementary Table S5. Sequences and limit of detection for HIV Flow-FISH assays, Supplementary Table S6. Sequences and limit of detection for SARS-CoV-2 Flow-FISH assays.
