# Supplementary material for: Flow-FISH as a Tool for Studying Bacteria, Fungi and Viruses
Source: BioTech (Basel). 2021 Oct 11;10(4):21. doi: 10.3390/biotech10040021 (PMC9245478; doi:10.3390/biotech10040021)
Supplement: Supplementary file 1 [file biotech-10-00021-s001.zip › biotech-1360488-Supplementary.pdf]

BioTech

Supplementary Material

Flow-FISH as a Tool for Studying Bacteria, Fungi and Viruses

Julian J. Freen-van Heeren

Supplementary Table S1. Sequences and limit of detection for bacterial Flow-FISH assays.

ND indicates not determined.

Species	Probe sequence(s) *(5′->3′)*	Limit of detection	Reference(s)
Bacillus cereus	GCTGCCTCCCGTAGG<br>GCTGCCTCCCGTAGGAGT	10^3 CFU/mL	[1]
Bacteroides vulgatus	GCACTTAAGCCGACACCT<br>CCAATGTGGGGGACCTT	ND	[2]
Bifidobacterium longum	GATAGGACGCGACCCCAT<br>CATCCGGCATTACCACCC	ND	[2]
Carnobacterium spp	GTCAGGGGATGAGCAGTTAC	ND	[3]
Clostridium spp	TAAGCCTTCCACTGTAGG<br>TAATCCTTCAACTGCTGG	ND	[4]
Collinsella aerofaciens	GGTCGGTCTCTCAACCC	ND	[2]
Desulfovibrio gigas	T(C/A)CGCA(G/A)ACTCAT CCCCAAA	10^5 CFU/mL	[5]
Desulfobacter hydrogenophilus	T(C/A)CGCA(G/A)ACTCAT CCCCAAA	ND	[5]
Escherichia coli	GCTGCCTCCCGTAGGAGT	ND	[5,6]
	GCTGCCTCCCGTAGGAGT	10^3 cells/mL	[7]
	GCTGCCTCCCGTAGGAGT	ND	[5]
	GCTGCCTCCCGTAGGAGT<br>GCAGCCACCCGTAGGTGT<br>GCTGCCACCCGTAGGTGT	ND	[8]
	GCTGCCTCCCGTAGGAGT<br>CATTTCACCGCTACACCT	ND	[9]
	TATCTAATCCTGTTT	ND	[10]
	GCTGCCTCCCGTAGGAGT<br>CTTTTGCAACCCACT	ND	[2]
Faecalibacterium prausnitzii	CCTCTGCACTACTCAAGAAAAAC	ND	[2]
Klebsiella pneumoniae	TATCTAATCCTGTTT	ND	[10]
Lactobacillus brevis	TGTTGAAATCAGTGCAAG	ND	[3]
Pseudomonas spp	GCTGCCTCCCGTAGGAGT	ND	[6]
	CCTTCCTCCCAACTT	ND	[9]
	TATCTAATCCTGTTT	ND	[10]
	AAACCGATCGTAGTCCGGATC	ND	[11]
	GCTGCCTCCCGTAGGAGT	ND	[7]
Ruminococcus productus	GCTTCTTAGTCARGTACCG	ND	[2]
Salmonella spp	AATCACTTCACCTACGTG<br>TCGACTGACTTCAGCTCC	10^3 cells/mL	[12–14]

Supplementary Table S2. Sequences and limit of detection for fungal Flow-FISH assays.

NA indicates not available; ND indicates not determined.

Species	Probe sequence(s) *(5′->3′)*	Limit of detection	Reference(s)
Candida albicans	NA (proprietary kit)	ND	[15]
	GCCAAGGCTTATACTCGCT<br>GGCTCCGTCAGTGTAGC<br>AGATTTCCCAGACCTCTCG<br>CGGCCATAAAGACCTACCAAGCG<br>CCACCAGCAGTCCGTCGTG<br>CCAGTTCTAAGTTGATCGTTAAACGTGCCC	ND	[16]
Saccharomyces carlsbergensis	ACCAGACTTGCCCTCC	ND	[6]
Staphylococcus aureus	NA (proprietary kit)	ND	[17,18]
	GCTGCCTCCCGTAGGAGT	ND	[7]
Staphylococcus epidermidis	AATATATTATCCGGT	ND	[19]

Supplementary Table S3. Sequences and limit of detection for viral Flow-FISH assays.

BVDV, Bovine viral diarrhea virus; γHV, Gamma herpesviruses; KSHV, Kaposi's sarcoma–associated herpesvirus; SV, Sindbis virus; SVV, Simian varicella virus; YFV, Yellow fever virus. NA indicates not available; ND indicates not determined.

Species	Probe sequence(s) *(5′->3′)*	Limit of detection	Reference(s)
BVDV	NA (proprietary kit)	ND	[20,21]
Dengue virus	NA (proprietary kit)	ND	[22]
HCV	NA (proprietary kit)	ND	[23]
γHV	NA (proprietary kit)	ND	[24]
KSHV	ACAAATGCCACCTCACTTTGTCGC<br>CGCTGCTTTCCTTTCACATT<br>GTGAAGCGGCAGCCAAGGTGACTGG	ND	[25]
	NA (proprietary kit)	ND	[24]
Parvovirus B19	CGCCTGGAACACTGAAACCC<br>GAAACTGGTCTGCCAAAGGT	ND	[26]
	Random probe set (generated via random priming on a DNA template)	ND	[27]
Poliovirus	NA (proprietary kit)	ND	[22]
SV	TCTTCTTCCCACACAGCGATAC	ND	[28]
SVV	NA (proprietary kit)	ND	[29]
YFV	NA (proprietary kit)	ND	[30,31]
Zika virus	NA (proprietary kit)	ND	[22,32]

Supplementary Table S4. Sequences and limit of detection for EBV Flow-FISH assays.

NA indicates not available; ND indicates not determined.

**Probe sequence(s)** *(5′->3′)*	**Limit of detection**	**Reference(s)**
AGACACCGTCCTCACCACCCGGGACTTGTA<br>CCCGGCTGCCCCAGGACCTGGCGGCGGCGC<br>CCGGCGGCCGCCCGGCTGCCCCCGGAGCGC	~1 infected cell / 9.000 cells	[33]
AAACATGCGGACCACCAGCTGGTAC<br>AAGACGGCAGAAAGCAGAGTCTGGG<br>AACCACAGACACCGTCCTCACCACC<br>GTGGCTACAGCCACACACGTCTCCT<br>AAACCTCTAGGGCAGCGTAGGTCCT	5-10 genome copies/cell	[34]
NA (proprietary probe)	~1 infected cell / 10.000 cells	[35]
NA (proprietary kit)	10 genome copies/cell	[24]
NA (proprietary kit)	> 4 log copies EBV/mL	[36]
NA (proprietary probe)	>1 infected cell / 500 cells	[37]
AGACACCGTCCTCACCACCCGGGACTTGTA<br>CCCGGCTGCCCCAGGACCTGGCGGCGGCGC<br>CCGGCGGCCGCCCGGCTGCCCCCGGAGCGC	ND	[38]

Supplementary Table S5. Sequences and limit of detection for HIV Flow-FISH assays.

NA indicates not available; ND indicates not determined.

**Probe sequence(s)** *(5′->3′)*	**Limit of detection**	**Reference(s)**
Pool of 50 probes	ND	[39,40]
NA (not specified)	ND	[41]
NA (proprietary kit)	0.5 - 10 infected cell / $1*10^6$ cells	[42–53]
Pool of 48 probes	ND	[54]

Supplementary Table S6. Sequences and limit of detection for SARS-CoV-2 Flow-FISH assays.

ND indicates not determined.

**Probe sequence(s)** *(5′->3′)*	**Limit of detection**	**Reference(s)**
Pool of 40 probes	ND	[55]

References

1. LaFlamme, C.; Gendron, L.; Turgeon, N.; Filion, G.; Ho, J.; Duchaine, C. Rapid Detection of Germinating Bacillus Cereus Cells Using Fluorescent in Situ Hybridization. *Journal of Rapid Methods and Automation in Microbiology* **2008**, *17*, 80–102.
2. Rigottier-Gois, L.; Le Bourhis, A.G.; Gramet, G.; Rochet, V.; Doré, J. Fluorescent Hybridisation Combined with Flow Cytometry and Hybridisation of Total RNA to Analyse the Composition of Microbial Communities in Human Faeces Using 16S RRNA Probes. *FEMS Microbiology Ecology* **2003**, *43*, 237–245, doi:10.1016/S0168-6496(02)00416-6.
3. Connil, N.; Dousset, X.; Onno, B.; Pilet, M.F.; Breuil, M.F.; Montel, M.C. Enumeration of Carnobacterium Divergens V41, Carnobacterium Piscicola V1 and Lactobacillus Brevis LB62 by in Situ Hybridization-Flow Cytometry. *Letters in Applied Microbiology* **1998**, *27*, 302–306, doi:10.1046/j.1472-765X.1998.00429.x.
4. Jen, C.J.; Chou, C.H.; Hsu, P.C.; Yu, S.J.; Chen, W.E.; Lay, J.J.; Huang, C.C.; Wen, F.S. Flow-FISH Analysis and Isolation of Clostridial Strains in an Anaerobic Semi-Solid Bio-Hydrogen Producing System by Hydrogenase Gene Target. *Applied Microbiology and Biotechnology* **2007**, *74*, 1126–1134, doi:10.1007/s00253-006-0740-8.
5. Amann, R.I.; Binder, B.J.; Olson, R.J.; Chisholm, S.W.; Devereux, R.; Stahl, D.A. Combination of 16S RRNA-Targeted Oligonucleotide Probes with Flow Cytometry for Analyzing Mixed Microbial Populations. *Applied and Environmental Microbiology* **1990**, *56*, 1919–1925, doi:10.1128/aem.56.6.1919-1925.1990.
6. Wallner, G.; Amann, R.; Beisker, W. Optimizing Fluorescent in Situ Hybridization with RRNA-targeted Oligonucleotide Probes for Flow Cytometric Identification of Microorganisms. *Cytometry* **1993**, *14*, 136–143, doi:10.1002/cyto.990140205.
7. Gunasekera, T.S.; Veal, D.A.; Attfield, P. v. Potential for Broad Applications of Flow Cytometry and Fluorescence Techniques in Microbiological and Somatic Cell Analyses of Milk. *International Journal of Food Microbiology* **2003**, *85*, 269–279, doi:10.1016/S0168-1605(02)00546-9.
8. Manti, A.; Boi, P.; Amalfitano, S.; Puddu, A.; Papa, S. Experimental Improvements in Combining CARD-FISH and Flow Cytometry for Bacterial Cell Quantification. *Journal of Microbiological Methods* **2011**, *87*, 309–315, doi:10.1016/j.mimet.2011.09.003.
9. Liu, P.; Meagher, R.J.; Light, Y.K.; Yilmaz, S.; Chakraborty, R.; Arkin, A.P.; Hazen, T.C.; Singh, A.K. Microfluidic Fluorescence in Situ Hybridization and Flow Cytometry (MFlowFISH). *Lab on a Chip* **2011**, *11*, 2673–2679, doi:10.1039/c1lc20151d.
10. Huang, X.X.; Urosevic, N.; Inglis, T.J.J. Accelerated Bacterial Detection in Blood Culture by Enhanced Acoustic Flow Cytometry (AFC) Following Peptide Nucleic Acid Fluorescence in Situ Hybridization (PNA-FISH). *PLoS ONE* **2019**, *14*, 1–17, doi:10.1371/journal.pone.0201332.
11. Gunasekera, T.S.; Dorsch, M.R.; Slade, M.B.; Veal, D.A. Specific Detection of Pseudomonas Spp. in Milk by Fluorescence in Situ Hybridization Using Ribosomal RNA Directed Probes. *Journal of Applied Microbiology* **2003**, *94*, 936–945, doi:10.1046/j.1365-2672.2003.01930.x.
12. Bisha, B.; Brehm-Stecher, B.F. Combination of Adhesive-Tape-Based Sampling and Fluorescence in Situ Hybridization for Rapid Detection of Salmonella on Fresh Produce. *Journal of Visualized Experiments* **2010**, 1–5, doi:10.3791/2308.
13. Bisha, B.; Brehm-Stecher, B.F. Simple Adhesive-Tape-Based Sampling of Tomato Surfaces Combined with Rapid Fluorescence in Situ Hybridization for Salmonella Detection. *Applied and Environmental Microbiology* **2009**, *75*, 1450–1455, doi:10.1128/AEM.01944-08.

14. Bisha, B.; Brehm-Stecher, B.F. Flow-through Imaging Cytometry for Characterization of Salmonella Subpopulations in Alfalfa Sprouts, a Complex Food System. *Biotechnology Journal* **2009**, *4*, 880–887, doi:10.1002/biot.200800360.
15. Trnovsky, J.; Merz, W.; Della-Latta, P.; Wu, F.; Arendrup, M.C.; Stender, H. Rapid and Accurate Identification of Candida Albicans Isolates by Use of PNA FISHFlow. *Journal of Clinical Microbiology* **2008**, *46*, 1537–1540, doi:10.1128/JCM.00030-08.
16. Bisha, B.; Kim, H.J.; Brehm-Stecher, B.F. Improved DNA-FISH for Cytometric Detection of Candida Spp. *Journal of Applied Microbiology* **2011**, *110*, 881–892, doi:10.1111/j.1365-2672.2011.04936.x.
17. Hartmann, H.; Stender, H.; Schäfer, A.; Autenrieth, I.B.; Kempf, V.A.J. Rapid Identification of Staphylococcus Aureus in Blood Cultures by a Combination of Fluorescence in Situ Hybridization Using Peptide Nucleic Acid Probes and Flow Cytometry. *Journal of Clinical Microbiology* **2005**, *43*, 4855–4857, doi:10.1128/JCM.43.9.4855-4857.2005.
18. Shrestha, N.K.; Scalera, N.M.; Wilson, D.A.; Brehm-Stecher, B.; Procop, G.W. Rapid Identification of Staphylococcus Aureus and Methicillin Resistance by Flow Cytometry Using a Peptide Nucleic Acid Probe. *Journal of Clinical Microbiology* **2011**, *49*, 3383–3385, doi:10.1128/JCM.01098-11.
19. Azevedo, N.F.; Jardim, T.; Almeida, C.; Cerqueira, L.; Almeida, A.J.; Rodrigues, F.; Keevil, C.W.; Vieira, M.J. Application of Flow Cytometry for the Identification of Staphylococcus Epidermidis by Peptide Nucleic Acid Fluorescence in Situ Hybridization (PNA FISH) in Blood Samples. *Antonie van Leeuwenhoek, International Journal of General and Molecular Microbiology* **2011**, *100*, 463–470, doi:10.1007/s10482-011-9595-9.
20. Falkenberg, S.M.; Dassanayake, R.P.; Neill, J.D.; Ridpath, J.F. Improved Detection of Bovine Viral Diarrhea Virus in Bovine Lymphoid Cell Lines Using PrimeFlow RNA Assay. *Virology* **2017**, *509*, 260–265, doi:10.1016/j.virol.2017.06.032.
21. Silveira, S.; Falkenberg, S.M.; Dassanayake, R.P.; Walz, P.H.; Ridpath, J.F.; Canal, C.W.; Neill, J.D. In Vitro Method to Evaluate Virus Competition between BVDV-1 and BVDV-2 Strains Using the PrimeFlow RNA Assay. *Virology* **2019**, *536*, 101–109, doi:10.1016/j.virol.2019.07.029.
22. Abernathy, E.; Mateo, R.; Majzoub, K.; van Buuren, N.; Bird, S.W.; Carette, J.E.; Kirkegaard, K. Differential and Convergent Utilization of Autophagy Components by Positive-Strand RNA Viruses. *PLoS Biology* **2019**, *17*, 1–28, doi:10.1371/journal.pbio.2006926.
23. van Buuren, N.; Kirkegaard, K. Detection and Differentiation of Multiple Viral RNAs Using Branched DNA FISH Coupled to Confocal Microscopy and Flow Cytometry. *BioProtocol* **2018**, *8*, 3058, doi:10.21769/BioProtoc.3058.Detection.
24. Oko, L.M.; Kimballid, A.K.; Kasparid, R.E.; Knoxid, A.N.; Coleman, C.B.; Rochford, R.; Chang, T.; Alderete, B.; van Dyk, L.F.; Clambey, E.T. Multidimensional Analysis of Gammaherpesvirus RNA Expression Reveals Unexpected Heterogeneity of Gene Expression. *PLoS Pathogens* **2019**, *15*, 1–29, doi:10.1371/journal.ppat.1007849.
25. Borah, S.; Nichols, L.A.; Hassman, L.M.; Kedes, D.H.; Steitz, J.A. Tracking Expression and Subcellular Localization of RNA and Protein Species Using High-Throughput Single Cell Imaging Flow Cytometry. *Rna* **2012**, *18*, 1573–1579, doi:10.1261/rna.033126.112.
26. Manaresi, E.; Bua, G.; Bonvicini, F.; Gallinella, G. A Flow-FISH Assay for the Quantitative Analysis of Parvovirus B19 Infected Cells. *Journal of Virological Methods* **2015**, *223*, 50–54, doi:10.1016/j.jviromet.2015.07.013.
27. Bua, G.; Manaresi, E.; Bonvicini, F.; Gallinella, G. Parvovirus B19 Replication and Expression in Differentiating Erythroid Progenitor Cells. *PLoS ONE* **2016**, *11*, 1–19, doi:10.1371/journal.pone.0148547.

28. Robertson, K.L.; Verhoeven, A.B.; Thach, D.C.; Chang, E.L. Monitoring Viral RNA in Infected Cells with LNA Flow-FISH. *Rna* **2010**, *16*, 1679–1685, doi:10.1261/rna.2016410.
29. Mahalingam, R.; Kaufer, B.B.; Ouwendijk, W.J.D.; Verjans, G.M.G.M.; Coleman, C.; Hunter, M.; Das, A.; Palmer, B.E.; Clambey, E.; Nagel, M.A.; et al. Attenuation of Simian Varicella Virus Infection by Enhanced Green Fluorescent Protein in Rhesus Macaques. *Journal of Virology* **2018**, *92*, doi:10.1128/jvi.02253-17.
30. Douam, F.; Hrebikova, G.; Albrecht, Y.E.S.; Sellau, J.; Sharon, Y.; Ding, Q.; Ploss, A. Single-Cell Tracking of Flavivirus RNA Uncovers Species-Specific Interactions with the Immune System Dictating Disease Outcome. *Nature Communications* **2017**, *8*, doi:10.1038/ncomms14781.
31. Sinigaglia, L.; Gracias, S.; Décembre, E.; Fritz, M.; Bruni, D.; Smith, N.; Herbeuval, J.P.; Martin, A.; Dreux, M.; Tangy, F.; et al. Immature Particles and Capsid-Free Viral RNA Produced by Yellow Fever Virus-Infected Cells Stimulate Plasmacytoid Dendritic Cells to Secrete Interferons. *Scientific Reports* **2018**, *8*, 1–15, doi:10.1038/s41598-018-29235-7.
32. McDonald, E.M.; Duggal, N.K.; Ritter, J.M.; Brault, A.C. Infection of Epididymal Epithelial Cells and Leukocytes Drives Seminal Shedding of Zika Virus in a Mouse Model. *PLoS Neglected Tropical Diseases* **2018**, *12*, 1–22, doi:10.1371/journal.pntd.0006691.
33. Crouch, J.; Leitenberg, D.; Smith, B.R.; Howe, J.G. Epstein-Barr Virus Suspension Cell Assay Using in Situ Hybridization and Flow Cytometry. *Cytometry* **1997**, *29*, 50–57, doi:10.1002/(SICI)1097-0320(19970901)29:1<50::AID-CYTO5>3.0.CO;2-H.
34. Stowe, R.P.; Cubbage, M.L.; Sams, C.F.; Pierson, D.L.; Barrett, A.D.T. Detection and Quantification of Epstein-Barr Virus EBER1 in EBV-Infected Cells by Fluorescent in Situ Hybridization and Flow Cytometry. *Journal of Virological Methods* **1998**, *75*, 83–91, doi:10.1016/S0166-0934(98)00104-9.
35. Kimura, H.; Miyake, K.; Yamauchi, Y.; Nishiyama, K.; Iwata, S.; Iwatsuki, K.; Gotoh, K.; Seiji, K.; Ito, Y.; Nishiyama, Y. Identification of Epstein-Barr Virus (EBV)-Infected Lymphocyte Subtypes by How Cytometric in Situ Hybridization in EBV-Assodated Lymphoproliferative Diseases. *Journal of Infectious Diseases* **2009**, *200*, 1078–1087, doi:10.1086/605610.
36. Fournier, B.; Boutboul, D.; Bruneau, J.; Miot, C.; Boulanger, C.; Malphettes, M.; Pellier, I.; Dunogué, B.; Terrier, B.; Suarez, F.; et al. Rapid Identification and Characterization of Infected Cells in Blood during Chronic Active Epstein-Barr Virus Infection. *Journal of Experimental Medicine* **2020**, *217*, doi:10.1084/JEM.20192262.
37. Kawabe, S.; Ito, Y.; Gotoh, K.; Kojima, S.; Matsumoto, K.; Kinoshita, T.; Iwata, S.; Nishiyama, Y.; Kimura, H. Application of Flow Cytometric in Situ Hybridization Assay to Epstein-Barr Virus-Associated T/Natural Killer Cell Lymphoproliferative Diseases. *Cancer Science* **2012**, *103*, 1481–1488, doi:10.1111/j.1349-7006.2012.02305.x.
38. Bernasconi, M.; Ueda, S.; Krukowski, P.; Bornhauser, B.C.; Ladell, K.; Dorner, M.; Sigrist, J.A.; Campidelli, C.; Aslandogmus, R.; Alessi, D.; et al. Early Gene Expression Changes by Epstein-Barr Virus Infection of B-Cells Indicate CDKs and Survivin as Therapeutic Targets for Post-Transplant Lymphoproliferative Diseases. *International Journal of Cancer* **2013**, *133*, 2341–2350, doi:10.1002/ijc.28239.
39. Wilburn, K.M.; Mwandumba, H.C.; Jambo, K.C.; Boliar, S.; Solouki, S.; Russell, D.G.; Gludish, D.W. Heterogeneous Loss of HIV Transcription and Proviral DNA from 8E5/LAV Lymphoblastic Leukemia Cells Revealed by RNA FISH:FLOW Analyses. *Retrovirology* **2016**, *13*, doi:10.1186/s12977-016-0289-2.
40. Jambo, K.C.; Banda, D.H.; Kankwatira, A.M.; Sukumar, N.; Allain, T.J.; Heyderman, R.S.; Russell, D.G.; Mwandumba, H.C. Small Alveolar Macrophages Are Infected Preferentially by HIV and Exhibit Impaired Phagocytic Function. *Mucosal Immunology* **2014**, *7*, 1116–1126, doi:10.1038/mi.2013.127.

41. Hanley, M.B.; Lomas, W.; Mittar, D.; Maino, V.; Park, E. Detection of Low Abundance RNA Molecules in Individual Cells by Flow Cytometry. *PLoS ONE* **2013**, *8*, doi:10.1371/journal.pone.0057002.
42. Pardons, M.; Baxter, A.E.; Massanella, M.; Pagliuzza, A.; Fromentin, R.; Dufour, C.; Leyre, L.; Routy, J.P.; Kaufmann, D.E.; Chomont, N. Single-Cell Characterization and Quantification of Translation-Competent Viral Reservoirs in Treated and Untreated HIV Infection. *PLoS Pathogens* **2019**, *15*, 1–28, doi:10.1371/journal.ppat.1007619.
43. Baxter, A.E.; Niessl, J.; Fromentin, R.; Richard, J.; Porichis, F.; Massanella, M.; Brassard, N.; Alsahafi, N.; Routy, J.-P.; Finzi, A.; et al. Multiparametric Characterization of Rare HIV-Infected Cells Using an RNA-Flow FISH Technique. *Nature Protocols* **2017**, *12*, 2029–2049, doi:10.1038/nprot.2017.079.
44. Baxter, A.E.; Niessl, J.; Fromentin, R.; Richard, J.; Porichis, F.; Charlebois, R.; Massanella, M.; Brassard, N.; Alsahafi, N.; Delgado, G.G.; et al. Single-Cell Characterization of Viral Translation-Competent Reservoirs in HIV-Infected Individuals. *Cell Host and Microbe* **2016**, *20*, 368–380, doi:10.1016/j.chom.2016.07.015.
45. Niessl, J.; Baxter, A.E.; Morou, A.; Brunet-Ratnasingham, E.; Sannier, G.; Gendron-Lepage, G.; Richard, J.; Delgado, G.G.; Brassard, N.; Turcotte, I.; et al. Persistent Expansion and Th1-like Skewing of HIV-Specific Circulating T Follicular Helper Cells during Antiretroviral Therapy. *EBioMedicine* **2020**, *54*, doi:10.1016/j.ebiom.2020.102727.
46. Bertram, K.M.; Botting, R.A.; Baharlou, H.; Rhodes, J.W.; Rana, H.; Graham, J.D.; Patrick, E.; Fletcher, J.; Plasto, T.M.; Truong, N.R.; et al. Identification of HIV Transmitting CD11c+ Human Epidermal Dendritic Cells. *Nature Communications* **2019**, *10*, doi:10.1038/s41467-019-10697-w.
47. Richard, J.; Prévost, J.; Baxter, A.E.; Bredow, B. von; Ding, S.; Medjahed, H. Uninfected Bystander Cells Impact the Measurement of HIV- Specific Antibody-Dependent Cellular Cytotoxicity Responses. *mBio* **2018**, *9*, e00358-18.
48. Rao, S.; Amorim, R.; Niu, M.; Temzi, A.; Mouland, A.J. The RNA Surveillance Proteins UPF1, UPF2 and SMG6 Affect HIV-1 Reactivation at a Post-Transcriptional Level. *Retrovirology* **2018**, *15*, 1–20, doi:10.1186/s12977-018-0425-2.
49. Grau-Expósito, J.; Serra-Peinado, C.; Miguel, L.; Navarro, J.; Curran, A.; Burgos, J.; Ocaña, I.; Ribera, E.; Torrella, A.; Planas, B.; et al. A Novel Single-Cell FISH-Flow Assay Identifies Effector Memory CD4+ T Cells as a Major Niche for HIV-1 Transcription in HIV-Infected Patients. *mBio* **2017**, *8*, 1–18, doi:10.1128/mBio.00876-17.
50. Grau-Expósito, J.; Luque-Ballesteros, L.; Navarro, J.; Curran, A.; Burgos, J.; Ribera, E.; Torrella, A.; Planas, B.; Badía, R.; Martin-Castillo, M.; et al. Latency Reversal Agents Affect Differently the Latent Reservoir Present in Distinct CD4+ T Subpopulations. *PLoS Pathogens* **2019**, *15*, 1–23, doi:10.1371/journal.ppat.1007991.
51. Real, F.; Capron, C.; Sennepin, A.; Arrigucci, R.; Zhu, A.; Sannier, G.; Zheng, J.; Xu, L.; Massé, J.M.; Greffe, S.; et al. Platelets from HIV-Infected Individuals on Antiretroviral Drug Therapy with Poor CD4+ T Cell Recovery Can Harbor Replication-Competent HIV despite Viral Suppression. *Science Translational Medicine* **2020**, *12*, 1–12, doi:10.1126/scitranslmed.aat6263.
52. Rao, S.; Lungu, C.; Crespo, R.; Steijaert, T.H.; Gorska, A.; Palstra, R.J.; Prins, H.A.B.; van Ijcken, W.; Mueller, Y.M.; van Kampen, J.J.A.; et al. Selective Cell Death in HIV-1-Infected Cells by DDX3 Inhibitors Leads to Depletion of the Inducible Reservoir. *Nature Communications* **2021**, *12*, 2475, doi:10.1038/s41467-021-22608-z.
53. Martrus, G.; Niehrs, A.; Cornelis, R.; Rechtien, A.; García-Beltran, W.; Lütgehetmann, M.; Hoffmann, C.; Altfeld, M. Kinetics of HIV-1 Latency Reversal Quantified on the Single-Cell Level Using a Novel Flow-Based Technique. *Journal of Virology* **2016**, *90*, 9018–9028, doi:10.1128/JVI.01448-16.

54. Abdel-Mohsen, M.; Kuri-Cervantes, L.; Grau-Exposito, J.; Spivak, A.M.; Nell, R.A.; Tomescu, C.; Vadrevu, S.K.; Giron, L.B.; Serra-Peinado, C.; Genescà, M.; et al. CD32 Is Expressed on Cells with Transcriptionally Active HIV but Does Not Enrich for HIV DNA in Resting T Cells. *Science Translational Medicine* **2018**, *10*, 30–35, doi:10.1126/scitranslmed.aar6759.
55. Zhang, Y.; Guo, R.; Kim, S.H.; Shah, H.; Zhang, S.; Liang, J.H.; Fang, Y.; Gentili, M.; Leary, C.N.O.; Elledge, S.J.; et al. SARS-CoV-2 Hijacks Folate and One-Carbon Metabolism for Viral Replication. *Nature Communications* **2021**, *12*, 1–11, doi:10.1038/s41467-021-21903-z.
